# Advancing Solar Energy for Primary Healthcare in Developing Nations: Addressing Current Challenges and Enabling Progress Through UNICEF and Collaborative Partnerships

**DOI:** 10.7759/cureus.51571

**Published:** 2024-01-03

**Authors:** Lokesh Sharma, Jaivardhan Singh, Ranjit Dhiman, Daniel R Vargas Nunez, Antoinette Eleonore Ba, Krupal J Joshi, Bhautik Modi, Abhishek Padhi, J. K Tandon, Michelle Seidel

**Affiliations:** 1 Public Health and Management, Jaipur National University, Namibia, ZAF; 2 Health Facilities Solar Electrification, United Nations Children's Fund (UNICEF), Copenhagen, DNK; 3 Public Health and Management, United Nations Children's Fund (UNICEF), Copenhagen, DNK; 4 Public Health and Management, United Nations Children's Fund (UNICEF), Nairobi, KEN; 5 Community and Family Medicine, All India Institute of Medical Sciences, Rajkot, IND; 6 Microbiology, All India Institute of Medical Sciences, Raipur, IND; 7 Public Health and Management, Jaipur National University, Jaipur, IND

**Keywords:** low- and middle-income countries (lmics), global health, renewable energy, sustainable development goals (sdgs), international aid organizations, healthcare facilities, solar energy

## Abstract

This comprehensive document explores the intersection of Sustainable Development Goals (SDGs) and the global transition to renewable energy, with a particular focus on solar energy. The text emphasizes the critical role of reliable and sustainable energy, especially solar power, in achieving health-related SDGs, particularly in low- and middle-income countries (LMICs). It discusses the challenges faced by healthcare facilities in these regions, emphasizing the importance of uninterrupted electricity for critical medical equipment and services. The document highlights the increasing significance of solar energy globally and its potential to address challenges in the healthcare sector.

The International Energy Agency's (IEA) estimation that solar photovoltaic (PV) energy has become the cheapest source of electricity is discussed, along with the World Bank's active role in supporting solar energy projects in developing countries. The document presents the current status of solarization, emphasizing the exponential growth of solar capacity and generation. It also discusses global initiatives such as Mission Innovation and the contribution of various international aid organizations, including Sustainable Energy for All (SEforALL), Power Africa, Lighting Global, SolarAid, UNDP - Solar for Health (S4H), and the World Bank.

A significant portion of the document focuses on the role of solar energy in healthcare, detailing successful solarization projects in India, sub-Saharan Africa, and other regions. It addresses the challenges of implementing solar PV projects in healthcare facilities, emphasizing the importance of maintenance and proper management. The document also provides insights into the contributions of United Nations Children's Fund (UNICEF) in advancing solar-powered health systems, emphasizing its support to over 80 countries in solarization and off-grid energy solutions for healthcare.

In conclusion, this article emphasizes the need for collaboration among international aid organizations, governments, and development partners to ensure universal access to reliable and sustainable electricity, particularly in healthcare facilities. It underscores the importance of long-term planning, sustainability, innovative business models, and awareness campaigns to achieve scalable and impactful results in the intersection of solar energy and healthcare delivery.

## Introduction and background

The Sustainable Development Goals (SDG) are a collection of 17 interlinked global goals designed to be a "shared blueprint for peace and prosperity for people and the planet, now and into the future". Among these, SDG 13 is to take urgent action to combat climate change and its impacts [1]. As climate change affects every country on every continent, it disrupts national economies and affects lives. It is expected that approximately 2,50,000 additional deaths per year due to climate change between the years 2030 and 2050. An increase in access to clean, modern, and affordable energy services is advocated in SDG 7 [2]. To ensure healthy lives and promote well-being, an uninterrupted supply of clean, modern, and affordable energy is required, as mentioned in SDG 7. United Nations Children's Fund (UNICEF) has pledged to fulfill the objectives of SDG 3 and SDG 6 regarding children and intends to aid in accomplishing SDG 7 and SDG 13 by implementing programmatic interventions. This aims to ensure providing climate-resilient, environmentally sustainable infrastructure of primary health centers with access to affordable, reliable, sustainable, and modern energy for all. Electricity enables the functionality of vital medical equipment, information and communication technologies, and essential services, including safe delivery, neonatal care, storage of vaccines and essential PHC commodities, lighting, space conditioning, provision of clean water, and proper sanitation.

Most healthcare facilities (HCF) in low- and middle-income countries (LMICs) have inadequate ensuring environments for delivering quality health services and the safety and well-being of healthcare workers. An estimated 50% of HCFs lack piped water on-premise, 33% lack improved sanitation facilities on the facility premises, 39% lack soap for handwashing, 39% lack adequate infectious waste disposal, 73% lack sterilization equipment, 74% lack guidelines for standard precautions, and 59% lack reliable electricity [3]. Around 12% of healthcare facilities lack any access to electricity in South Asia. Around 15% of healthcare facilities lack any access to electricity in sub-Saharan Africa. Only 50% of hospitals in sub-Saharan Africa report reliable electricity access. Around 1 billion are estimated to be served by healthcare facilities with no or unreliable electricity supply worldwide [4].

In recent decades, exponential economic development, along with population growth, have led to an increasing demand for energy globally. However, it is the developing countries that play a key role in this phenomenon. According to the International Energy Agency's report of 2017, "World energy consumption is expected to grow from 575 quadrillion British thermal units (Btu) in 2015 to 663 quadrillion Btu by 2030; mostly this energy demand is predicted to come from developing countries". Considering this prediction, it is safe to say that developing countries are destined to experience a significant increase in both production and consumption of energy in the near future [5-8]. Thus, the transition toward renewable energy, including solar energy, thermal energy, hydroelectric energy, etc., in developing countries, which appears to be the most viable way to achieve this, is going to play a critical role in achieving environmental sustainability at the global level [9].

The current review identifies the current status of solar energy utilization for primary healthcare in developing countries and the challenges faced and efforts made by different international aid organisations.

## Review

Achieving zero greenhouse gas emissions is crucial to prevent a climate disaster. This requires us to accelerate and optimize the use of existing renewable energy tools such as solar and wind power. Additionally, it is necessary to develop and implement innovative technologies that can help us reach our goal [10].

In developing countries, renewable energy, such as hydropower and wind power, has a well-established history in electricity generation [11]. Despite this, the adoption of solar energy remains limited in these regions. The residential sector predominantly relies on wood, pellets, and charcoal for cooking and heating, contributing to concerns about carbon emissions and their associated effects on climate change, land use, environment, health, energy efficiency, and social justice. Addressing these issues is crucial for promoting sustainable and inclusive energy practices in these nations [11-13]. 

In this context, the adoption of solar energy offers a multitude of advantages, making it one of the model choices for sustainable energy in developing countries, most of which are geographically suited to receive optimal sunlight, making solar photovoltaic systems ideal for maximizing the potential of energy production. Thus, solar energy is a practical, reliable, and cost-effective method of sustainable energy generation in these settings.

The International Energy Agency (IEA) estimates that solar photovoltaic (PV) energy has become the cheapest source of electricity in many parts of the world, including in developing countries. According to the IEA's 2020 World Energy Outlook report, The cost of solar photovoltaic (PV) electricity has seen a dramatic decrease, approximately 80% since 2010, positioning it as a cheaper alternative to coal and gas in many regions. This shift is attributed to advancements in technology, increased efficiency, and supportive regulatory environments. Studies indicate that solar PV now competes favorably with traditional energy sources, with its cost decline catalyzing the integration of sustainable technologies and enabling the significant potential for economic growth, particularly in countries like China. Furthermore, the transition towards solar energy is not only environmentally strategic but also economically viable, as it now constitutes the majority of new generation capacity installed globally [14].

The World Bank has been actively supporting the development of solar energy projects in developing countries. The World Bank has financed more than 1,000 solar projects in over 100 countries, with a total capacity of more than 16 GW. The bank notes that solar power is particularly well-suited to many developing countries because it can be deployed quickly, does not require large upfront investments, and can be scaled up as demand grows [15].

Current status of solarization

Solar energy has been used not only for household and commercial units, but also for powering automobiles. In the last two decades, solar energy implementation models have become progressively efficient, cost-effective, and versatile. 

In 1994, Shell International Petroleum (London, UK) predicted that by 2050, half of the world's energy production would be derived from renewable sources, particularly solar energy. By 1999, renewable resources met 13% of global energy demand, and solar electric installations experienced substantial growth from 200MW in 1999 to 427MW in 2002. Despite this upward trajectory, solar energy still represented only about 0.1% of the world's primary energy demand until 2010 [16].

The subsequent decade witnessed a remarkable surge in solar capacity, driven by decreasing module prices and national commitments to reduce greenhouse gas emissions and enhance electricity accessibility [17]. While early solar installations were spearheaded by Europe, the US, and Japan, recent years have seen developing countries, notably China and India, take the lead in the solar sector [12]. In 2016, China alone added approximately 27GW of total solar capacity, constituting about 40% of the global solar capacity installed that year. Although per-capita installations in Africa remained modest, the region demonstrated noteworthy growth. According to BNEF, more than 1.5 million households in Africa utilized solar home systems in 2017, with mobile-money-enabled financing plans contributing to an almost 300% increase in cumulative installations compared to 2015 [16,17].

Solar capacity and generation have been increasing globally over the past decade. According to an International Renewable Energy Agency (IRENA) report, in 2021, global solar PV generation rose by 179 TWh, and solar capacity reached 849 GW, accounting for 3.6% of the world's energy generation. China leads the world with 392 GW of solar capacity, while the USA, Japan, Germany, and India follow with 135.7 GW, 84.9 GW, 66.5 GW, and 63.3 GW, respectively. Solar power is becoming an increasingly significant contributor to the electricity mix of many countries [18].

Nevertheless, as the new decade commenced, solar energy comprised merely 5% of overall capacity and contributed only 1.3% to global electricity generation. Despite these relatively modest figures, the substantial growth observed in recent years can be attributed to national policies and a noteworthy reduction in photovoltaic module prices, dropping more than threefold [19].

Mission Innovation is a global initiative with 25 participating countries and the EU aimed at accelerating clean energy innovation to address climate change. Its objective is to achieve global access to affordable, reliable, sustainable, and modern energy services by 2030, in line with the UN's Sustainable Development Goal 7. The initiative aims to double the public investment in clean energy R&D by member countries by 2025 to accelerate the development and deployment of innovative clean energy technologies to reduce greenhouse gas emissions and mitigate climate change.

In May 2022, the International Energy Agency reported the addition of 295 gigawatts of new renewable power capacity in 2021 despite supply chain bottlenecks, construction delays, and inflation in the prices of raw materials. Solar energy will account for 60% of renewable power, surpassing wind and hydropower [20].

UNICEF has procured and installed more than 80,000 solar refrigerators globally for the immunization program. There are a lot of different examples from various countries using solar energy [20]. 

Solar energy for healthcare

Healthcare facilities need a constant and unhindered supply of energy to ensure that all essential health services are offered to meet the demand. Delicate lifesaving machinery, including life monitors, resuscitation and life support systems, complex diagnostic equipment and scanners, and different departments, including emergency response units, critical care units, and even routine outpatient care, will be rendered handicapped without uninterrupted electricity.

Health facilities should give priority to critical loads, focusing on specific requirements like lighting in surgical theatres, maintaining cold chains for vaccines and lab samples, and ensuring uninterrupted power for essential medical equipment such as oxygen concentrators and ventilators. By thoroughly considering these critical loads, systems can be strategically designed to ensure a constant and reliable power supply for these crucial elements.

An energy-intensive utility like hospitals can tremendously benefit from solar energy for reliable, round-the-clock, economical, and waste-free sources of energy. In 2017, recognizing the importance of this requirement, the Indian Council of Medical Research (ICMR) entered into a Memorandum of Understanding (MoU) with The Council on Energy, Environment, and Water (CEEW). This collaboration aimed to initiate the 'Solar for Healthcare' project, conducting pilots across three states in India. The project involved the installation of solar systems at selected Primary Health Centers (PHCs), followed by comprehensive monitoring and evaluation to assess the impact of enhanced electricity access on healthcare delivery. Another project under this initiative included a proposal by the Department of Science and Technology to fund the installation of solar systems in health facilities across Odisha.

There are quite a few examples highlighting the success story of solarization of health facilities in India. A tertiary care hospital located in a rural area of India, catering to over 450 neighboring villages, continued providing services from essential healthcare to intensive care without energy crisis during the COVID-19 pandemic due to an elegant forethought of harnessing solar power, shifting from a three-phase grid to a 10kWp solar photovoltaic system [21-23]. Comparable initiatives were implemented in sub-Saharan Africa to tackle the issue of insufficient electrification in healthcare facilities. The World Bank undertook a study encompassing all primary public health clinics in Liberia, revealing that access to electricity rose from 54% in 2011 to 62% in 2012. This improvement was largely attributed to the installation of solar photovoltaic (PV) systems [17, 22].

Challenges of implementing solar PV projects

A solar photovoltaic (PV) system, which is not connected to the main power grid, generates energy and can be used to charge a battery bank and supply electricity to a building and/or specific appliances. Solar photovoltaic (PV) systems suitable for primary health care facilities typically vary in size from 1500 Wp to 20 kWp. System capacities exceeding 20 kWp are commonly found in larger health facilities, particularly those already connected to the grid. The average size of a primary healthcare center is approximately 5 kW [23]. Despite solar PV systems generating electricity in direct current (DC), most medical appliances operate on alternating current (AC). To accommodate this, solar PV systems are equipped with inverters, enabling the conversion of DC to AC. This feature broadens the range of equipment that these systems can power. The drop in solar panels and battery costs, along with significant improvements in battery technology worldwide, is projected to continue, making it increasingly more competitive with other renewable energy technologies, as well as the grid. The solar systems can operate for more than 25 years for an output of 80% capacity if adequate operations and maintenance (O&M) and replacements are carried out. The need for O&M and replacement needs can be brought down if good quality equipment is purchased. In any case, many PV systems could become inoperative after three to five years (or less) if proper maintenance and repair services are not provided.

Sustaining energy systems necessitates regular maintenance to uphold their long-term functionality and reliability. Without a proper maintenance routine, which includes both preventive and corrective maintenance, PV systems performance deteriorates and, over time, could result in damage and failures. The above challenge can be addressed if a professional is conducting adequate maintenance actions. However, in a developing country's context, once the solar equipment is handed over to the national partners, then in most of the instances, the national partners do not have adequate human and financial resources to ensure adequate management of operations and maintenance services. This results in premature failure of solar equipment. In addition, in a lot of cases, the handover process and the roles and responsibilities are not laid out between the different stakeholders involved during project planning and implementation. This leads to negligence of operation and maintenance activities as no agency has the mandate to carry it out.

This traditional model of completely relying on the national government agency to fully manage and fund the operation and maintenance contract over the long term has failed and has resulted in underutilization of the investment put into capital to purchase these solar systems.

Different international aid organizations working in the field of solar energy

Primary healthcare in many lower- and middle-income countries (LMICs) is dependent on crucial intervention from the various aid organizations. One key aspect of this is supporting the health care facility with access to reliable and sustainable electricity through the use of solar PV technology.

Various independent international organizations collaborate with the United Nations (UN), governmental leaders, private sectors, financial institutions, civil society, and philanthropies to accelerate progress toward achieving Sustainable Development Goal 7 (SDG7). This goal aims to ensure access to affordable, reliable, sustainable, and modern energy for all by 2030, aligning with the Paris Agreement on Climate Change. These partnerships play a crucial role in supporting local governments and health facilities, enabling them to extend services to remote areas.

Sustainable Energy for All (SEforALL)

In 2021, SEforALL actively assisted 27 countries in their pursuit of SDG7 through a combination of implementation support, advocacy, advisory services, and research. SEforALL is currently aiding the Government of Nigeria in initiating the implementation of its Energy Transition Plan, which targets achieving net-zero emissions by 2060. The first wave of the Universal Energy Facility, a results-based financing facility managed by SEforALL, is expected to generate around 14,000 new electricity connections [22].

Power Africa

As a U.S. Government-led partnership, Power Africa brings together over 170 public and private sector partners to double access to electricity in sub-Saharan Africa. Since 2013, Power Africa-supported projects have contributed nearly 12,500 megawatts (MW) of cleaner and more reliable electricity, providing over 27 million new power connections for homes and businesses. Power Africa's ambitious goal is to add at least 30,000 MW and facilitate 60 million new connections by 2030 [24].

Lighting Global

Lighting Global is the World Bank Group's platform to support the sustainable growth of the international off-grid solar market as a means of rapidly increasing energy access to the 789 million people living without electricity. Lighting Global works with manufacturers, distributors, governments, and other development partners to build and grow the modern off-grid solar energy market. To date, nearly 180 million people have benefited from using Lighting Global quality verified solar products, and more than 59 million people are currently meeting their basic (Tier 1) electricity needs [25].

SolarAid

SolarAid is an international charity founded in 2006 to combat poverty and climate change. Through its social enterprise, SunnyMoney, it provides access to solar lights in Malawi and Zambia [26].

UNDP - Solar for Health (S4H)

Working in the field of Solar Energy since 2017, the United Nations Development Programme (UNDP) has initiated Solar for Health (S4H) interventions to support different countries and install solar PV systems in over 900 health centers and medical storage facilities.

World Bank - West Africa Regional Off-Grid Electricity Access Project (ROGEAP)

The World Bank has launched an effort under the ROGEAP to help Niger and Nigeria electrify health and education facilities [27].

Contribution of UNICEF in solar-powered health systems advancement

Sustainable Development Goals are interlinked directly and indirectly. To achieve Goal 3 and Goal 6, logistics and services are required. To ensure healthy lives and promote well-being, an uninterrupted supply of clean, modern, and affordable energy is required, as mentioned in SDG 7. UNICEF is committed to channeling the efforts to work towards SDG 3 and SDG 6, which can be improved by working on SDG 7, which aims to ensure access to affordable, reliable, sustainable, and modern energy for all.

UNICEF has expanded its support to over 80 countries by facilitating the adoption of solarization and off-grid energy solutions, particularly focusing on critical areas such as vaccine cold chains, electrification, and heating and cooling at health facilities and medical warehouses. The organization is actively engaged in providing technical assistance to offer evidence-based policy support, emphasizing the interconnections between health and renewable energy.

From 2016 to 2021, UNICEF successfully procured and delivered solar-powered vaccine cold chain equipment valued at over $333 million. Over 88,000 solar cold-chain vaccine fridges have been installed in healthcare facilities, primarily across Africa, since 2017. UNICEF's commitment to leveraging renewable energy for health, education, and water and sanitation hygiene is outlined in the publication titled "A Brighter Life for Every Child with Sustainable Energy." Highlighted initiatives in the document include the solarisation of 70 rural healthcare facilities in Syria from 2016 to 2021, and the scaling up of solar hybrid photovoltaic (PV) systems in Maharashtra, starting with 58 installations in 2009 and reaching 407 systems in 29 districts by 2015 [28].

In the 2020-2021 period, UNICEF implemented nearly 3000 solar-powered water systems (SPWS) in schools, healthcare facilities, and communities across 51 countries through the Global Solar Water Pumping Programme [29]. The organization played a vital role in mitigating drought risks by deploying solar-powered water pumping systems in Colombia and Mauritania. In the Gaza Strip, supported by funding from the European Union, UNICEF constructed and continues to manage the Southern Gaza Desalination Plant, ensuring safe water access for hospitals, factories, schools, households, and more.

By the end of 2021, UNICEF Malawi successfully installed 429 solar vaccine cold chain units, conducted energy demand assessments at over 50 healthcare facilities, and installed solar PV systems in 20 health facilities (with an additional five in progress in 2022). Notably, UNICEF Lebanon has also solarized 150 health facilities as part of its ongoing efforts.

## Conclusions

This review advocates the need to expand the facility of Solar energy to various health centers. The coverage of Solar Governments must lead to providing reliable and sustainable electricity for universal access, especially in crucial areas like primary healthcare facilities.

Ensuring the sustainability of the solar project is the major challenge; therefore, organizations should heavily focus on investing in sustainability and consider innovative business models for achieving scalability and faster impact for healthcare facilities beneficiaries.

Other challenges are the lack of robust policy and a supportive regulatory environment. Working in this direction lays the groundwork for international development organizations and partners to contribute effectively. Collaboration among aid organizations is crucial for coordinated solarization efforts, sharing experiences, and learning across countries, requiring long-term planning and formalized roles through MOUs. Results-based financing and risk mitigation, like default Escrow accounts, can ensure timely funds for companies meeting targets. Coordinated advocacy by development organizations can urge governments to prioritize healthcare and energy access, aligning with national energy goals and fostering local energy market growth to meet broader population needs.

## References

[1] (2023). 1 in 3 people globally do not have access to safe drinking water - UNICEF. https://www.who.int/news/item/18-06-2019-1-in-3-people-globally-do-not-have-access-to-safe-drinking-water-unicef-who.

[2] Nitsch J, Staib F (2003). Strategies for introducing renewable energies and the contribution of photovoltaics. Photovoltaics Guidebook for Decision-Makers.

[3] Cronk R, Bartram J (2018). Environmental conditions in health care facilities in low- and middle-income countries: coverage and inequalities. Int J Hyg Environ Health.

[4] (2023). Database on electrification of health-care facilities. https://www.who.int/data/gho/data/themes/database-on-electrification-of-health-care-facilities.

[5] Hangun B, Eyecioglu O, Beken M (2022). Investigating the energy production trends of countries and its relationship between economic development. 2022 11th International Conference on Renewable Energy Research and Application (ICRERA.

[6] Barykina Y, Chernykh A, Na B (2022). Energy production as a basis for sustainable development in the BRICS countries. IOP Conference Series: Earth and Environmental Science.

[7] Lee H-y, Jang KM, Kim Y (2020). Energy consumption prediction in Vietnam with an artificial neural network-based urban growth model. Energies.

[8] Fu Q, Álvarez-Otero S, Sial MS, Comite U, Zheng P, Samad S, Oláh J (2021). Impact of renewable energy on economic growth and CO2 emissions - evidence from BRICS countries. Processes.

[9] Yang S, Park S (2020). The effects of renewable energy financial incentive policy and democratic governance on renewable energy aid effectiveness. Energy Policy.

[10] Gates B (2023). My new climate book is finally here. https://www.gatesnotes.com/My-new-climate-book-is-finally-here.

[11] Solarin SA, Al-Mulali U, Gan GG, Shahbaz M (2018). The impact of biomass energy consumption on pollution: evidence from 80 developed and developing countries. Environ Sci Pollut Res Int.

[12] Thomas PJ, Rosenberg-Jansen S, Jenks A (2021). Moving beyond informal action: sustainable energy and the humanitarian response system. J Int Humanit Action.

[13] Vanegas Cantarero MM (2020). Of renewable energy, energy democracy, and sustainable development: a roadmap to accelerate the energy transition in developing countries. Energy Res Soc Sci.

[14] (2020). World energy outlook. https://www.iea.org/reports/world-energy-outlook-2020.

[15] (2023). World Bank. Solar power. https://www.worldbank.org/en/topic.

[16] Dornan M, Shah KU (2016). Energy policy, aid, and the development of renewable energy resources in Small Island Developing States. Energy Policy.

[17] Patlitzianas KD (2011). Solar energy in Egypt: significant business opportunities. Renew Energy.

[18] (2023). The top 5 solar countries in the world. https://ornatesolar.com/blog/the-top-5-solar-countries-in-the-world.

[19] Theron A (2017). How solar power can improve healthcare services: lessons from India. https://www.esi-africa.com/research-and-development/india-best-practice-solar-power-can-improve-healthcare-services/.

[20] Tesfaye T, Temesgen B, Mitiku S (2022). The effect of solar direct derive cold chain equipment on the performance of immunization services in health facilities of Ethiopia. J Health Manag.

[21] Concession L, Gupta DK, Deka P (2023). Solar Power: a rural Indian hospital's key to fighting COVID-19. https://www.wri.org/insights/solar-power-rural-indian-hospitals-key-fighting-covid-19.

[22] Pratiyuksha VN, Sundararaman K, Ranjitha K (2020). Development of renewable energy in India and challenges faced. 2020 International Conference on Power Energy, Control and Transmission Systems.

[23] (2023). Powering Primary Health Care through Solar in India. https://smartnet.niua.org/sites/default/files/resources/powering_primary_healthcare_through_solar_in_india_-_lessons_from_chhattisgarh.pdf.

[24] (2023). Annual report SE4All. https://www.seforall.org/system/files/2022-08/SEforALL%20Annual%20Report%202021_0.pdf.

[25] (2023). Power Africa. https://www.usaid.gov/powerafrica/annualreport.

[26] (2023). Lighting Global. https://www.lightingglobal.org/.

[27] (2023). Solar Aid. https://solar-aid.org/about-solaraid/.

[28] (2023). A brighter life for every child with sustainable energy. https://www.unicef.org/media/127626/file/A%20brighter%20life%20for%20every%20child%20with%20sustainable%20en.

[29] (2023). Solar-powered water systems. https://www.unicef.org/wash/solar-powered-water-systems.

